# Differential effects of 2-deoxy-D-glucose on in vitro expanded human regulatory T cell subsets

**DOI:** 10.1371/journal.pone.0217761

**Published:** 2019-06-06

**Authors:** Naoki Tanimine, Sharon K. Germana, Martin Fan, Keli Hippen, Bruce R. Blazar, James F. Markmann, Laurence A. Turka, Bhavana Priyadharshini

**Affiliations:** 1 Center for Transplantation Sciences, Department of Surgery, Massachusetts General Hospital, Boston, Massachusetts, United States of America; 2 Department of Pediatrics, Division of Hematology/Oncology and Blood and Marrow Transplantation, University of Minnesota Cancer Center, Minneapolis, Minnesota, United States of America; 3 Rheos Medicines, Boston, Massachusetts, United States of America; University of Iowa, UNITED STATES

## Abstract

Regulatory T cells (Tregs) are required for the maintenance of immune tolerance and adoptive Treg infusion therapy has become a promising approach to suppress immune responses in diseases such as autoimmunity and transplant rejection. However, one critical challenge of Treg therapy is the requirement of in vitro expansion of functionally stable Tregs while preventing either the contamination of T effector and/or emergence of unstable pathogenic Tregs. Recent studies showing distinct metabolic requirements of T effectors and Tregs suggest that manipulation of cell metabolism may be an attractive strategy to achieve this goal. Here we show that human thymically derived Tregs (tTregs) and in vitro induced Tregs (iTregs) from naive T cells engage glycolysis equivalently upon activation. However, inhibiting glucose metabolism via 2-deoxy-D-glucose (2DG) has distinct effects on each of these subsets. While 2DG treatment at the onset of activation significantly reduced the proliferation and expression of suppressive molecules such as ICOS and CTLA-4 in tTregs, its effect on FOXP3 expression was small. In contrast, 2DG treatment during iTreg induction modestly decreased their proliferation but strongly reduced both ICOS and FOXP3 expression. Importantly, both Treg subsets became insensitive to 2DG after day 3 post activation with little effect on either proliferation or FOXP3 expression while T conventional Th0 cells showed reduced proliferation under the same conditions. Moreover, 2DG treatment at day 3 did not impair the suppressive capabilities of Treg subsets. Collectively, these findings suggest that there is a distinct temporal requirement of glycolysis in each of the activated human Treg subsets and T conventional cells. Furthermore, 2DG treatment at the onset as a strategy to impair contaminating T effector cell proliferation is unfavorable for optimal Treg generation as well.

## Introduction

Regulatory T cells (Tregs) are critical for the prevention of autoimmunity [1] and for transplantation tolerance [2]. Given their therapeutic potential, clinical trials of Treg immunotherapy are already underway in patients with autoimmune diseases and recipients of stem cell and solid organ transplants[3]. Despite these advances, it remains challenging to manufacture sufficient numbers of functionally stable Tregs and optimize in vivo conditioning treatments, which highlight the issue of feasibility and safety with current approaches [4, 5].

Tregs are comprised of two distinct subsets. One is thymically derived Tregs (tTregs) while the other is comprised of peripherally induced Tregs (pTregs), that originate from naïve T cells and acquire forkhead box P3 (FOXP3) protein following stimulation [6]. In vitro induced Tregs (iTregs) (derived naïve T cells activated in the presence of anti-inflammatory cytokines such as transforming growth factor-beta (TGF-β)) are usually considered the in vitro counterpart of pTreg [6]. Epigenetic studies in tTregs and iTregs reveal that while there is full demethylation of the Treg specific demethylation region within *Foxp3* gene in tTregs, which confers functional stability, it is incompletely demethylated in iTregs, which potentially renders them susceptible to conversion into pathogenic T effectors [7]. Therefore, tTregs are considered by many as the more desirable candidates for Treg expansion protocols.

Studies in the field of immunometabolism have shown that different metabolic pathways dictate immune cell fate and function [8, 9]. iTregs generated in mouse models only minimally utilize glycolysis, a hallmark feature for T effectors, but instead rely upon fatty acid oxidation (FAO) for their differentiation [10, 11]. Moreover, murine FOXP3 can itself drive oxidative phosphorylation and reprogram the metabolism of T cells away from glycolysis [12, 13, 14]. The conditions, which can result in the hyperactivation of glycolytic metabolism such as inflammation (TLR2 signaling) and defects in autophagy (via deletion of ATG5/7), are often associated with the loss of FOXP3 in tTregs and their destabilization [14, 15, 16]. Although these findings suggest the potential to boost Tregs via inhibition of glucose metabolism, more recent studies suggest that glucose metabolism is in fact important for optimal Treg function. For instance, murine tTregs (in contrast to iTregs) depend upon glycolytic-lipogenesis for establishing their suppressive function via upregulation of (cytolytic T lymphocyte-associated antigen- 4 (CTLA-4) and inducible T cell costimulator (ICOS) [17]. Furthermore, freshly isolated ex vivo human Tregs engage both glycolysis and FAO upon activation [18] and human iTregs generated by a weak TCR stimulation, without exogenous TGF-β, actually require glycolysis for optimal expression of the FOXP3 exon2 splice variant, which is critical for their suppressive function [19]. Together these studies indicate that metabolic landscape of human Treg subsets and its connection with FOXP3 expression is distinct compared to the murine system.

In order to understand the glycolytic requirements of human Tregs subsets, here we directly compared the metabolic requirements of pre-existing human ex vivo Tregs (which are predominantly tTreg [20]) and iTregs at different stages of in vitro activation. We show that both human Treg subsets engage in glycolysis to a similar degree at day 3 post activation. 2-deoxy-D-glucose (2DG) treatment, that inhibits not only glycolysis but also pentose phosphate pathway, glycolytic oxidation and protein glycosylation, at either the onset or day 3 post activation revealed that although glycolysis is required for cell growth and proliferation in both subsets, it is predominantly required for optimal expression of inhibitory molecules such as CTLA4 and ICOS in tTregs and critical for the FOXP3 induction in iTregs during early activation. However, by day 3 post activation, both human Treg subsets are unaffected by 2DG for their FOXP3 expression and proliferation suggesting that glucose metabolism is dispensable during the late phase of Treg expansion. Particularly, 2DG treatment at the late phase doesn’t alter tTreg suppressive capacity in vitro. Interestingly, 2DG at day 3 post activation diminishes the proliferation of conventional Th0 cells indicating a differential requirement of glucose metabolism in these two subsets at the late phase of activation. These findings provide novel insights into the temporal metabolic requirements of human Treg subsets and suggest that there could be a crucial window of opportunity to selectively modulate glycolysis to control balance between Treg and T effectors.

## Material and methods

### Human Treg and naïve T cell isolation

This study was approved by Partners Human Research Committee (Protocol 2013P002335). Written form of the consent was obtained from all the participants. Normal donor apheresis units obtained from the Massachusetts General Hospital Blood Bank were processed for peripheral blood mononuclear cells (PBMCs) over a Ficoll-Paque PLUS gradient (GE Healthcare). Frozen cell aliquots were thawed and used (viability 90–99%) to enrich CD4^+^ T cells using the Human CD4 T cell enrichment Kit (Invitrogen). Cells were labeled with antibodies to CD4, CD25, CD127, CD45RA, and Fixable Live/Dead Aqua (Invitrogen) and sorted on FACS Fusion (BD Biosciences). Ex vivo Tregs and naïve T conventional (nTconv) cells were defined as CD4^+^CD25^+^CD127^low/-^ cells and CD4^+^CD25^-^CD127^+^CD45RA^+^, respectively (S1 Fig). Post sort purities were typically > 95%.

### Treg cultures

Ex vivo Tregs were cultured with 300IU/ml IL-2 (Proleukin; kindly provided by Bluestone lab, UCSF) and nTconv cells were cultured for iTreg induction with 300IU/ml IL-2, 5ng/ml TGF-β (Peprotech), and 10nM all trans retinoic acid (ATRA, Sigma) as previously described [21, 22]. nTconv cells were cultured with 300IU/ml IL-2 alone, called Th0 cells. Briefly, cells were activated with anti-CD3/anti-CD28 coated microbeads (11141D, Invitrogen) at a 1:1 ratio. On day 3, the culture volume was doubled with media. On day 5 and 7, cells were counted and media was replenished to a concentration of 2.5 x10^5^ cells/ml. IL-2 was added to a final concentration of 300IU/ml on day3, 5 and 7. 2DG (Sigma) was added for inhibiting glycolytic metabolism at indicated doses and time points.

### Flow cytometry

The following Abs were used for phenotypic analysis: Alexa Fluor 488-FOXP3 (PCH101), ICOS (C394.8A), PE-FOXP3 (D150/E4), CTLA4 (L3D10), PECy7-CD4 (RPA-T4), APC-CD25 (BC96), IFN gamma (4S.B3), APCCy7-CD45RA (HI100), BV421-CD127 (A019D5). For proliferation assay, Cell Trace Violet (Invitrogen) was used at 5uM according to manufacturer’s protocol. Proliferation index (PI) was calculated as mitotic events divided by precursor frequency [23]. For cytokine detection, cells are activated by 50ng/ml phorbol myristate acetate and 0.5ug/ml ionomycin in the presence of brefeldin A for 4 hours prior to staining. Intracellular staining was performed with Foxp3/Transcription Factor Staining Buffer Kit (Thermo Fisher) as manufacture protocol. Data was collected on a Navios (Beckmann Coulter), FACS Fusion, or FACS Verse (BD Biosciences) and analyzed using Flowjo (Tree Star).

### Metabolic assays

Real-time measurements of extracellular acidification rate (ECAR) and oxygen consumption rate (OCR) were estimated using an XFe-96 Extracellular Flux Analyzer (Agilent). Cells were plated in Cell Tak prepared XFe-96 plates at a concentration of 2 x 10^5^ cells/well. Glycolysis stress and mito-cell stress tests were conducted in XF media supplemented with 4mM L-glutamine and XF media supplemented with 10 mM glucose, 4 mM L-glutamine, and 5 mM sodium pyruvate, respectively.

### Quantitative PCR

cDNA was synthesized from total RNA with iScript Reverse Transcription Supermix (BIO-RAD). Quantitative PCR was performed on a Stratagene MX3005P with a SYBR Green detection system (RT^2^ SYBR Green ROX qPCR mastermix; Qiagen). Relative expression of genes was normalized to beta-actin (β-actin).

### In vitro Treg suppression assay

Suppression assays were performed with 2.5% FBS containing complete medium. Briefly tTregs and iTregs cultured for 7 days in the presence or absence of 2DG (0.5mM) that initiated from day 3 post activation. Treg subsets were rested in 50 IU/ml IL-2 containing medium for an additional 2 days prior to assay for 7 day culture product. Autologous total human CD4 T cell enriched from PBMCs were labeled with CFSE at 5uM for 5mins and used as responders. CFSE stained responders (50,000 cells) were cultured in a 96 well U-bottom plate in 200ul medium and stimulated by anti-CD3/CD28 microbeads at 1:5 ratio (bead: cell) in the presence or absence of suppressors at indicated responder to suppressor ratio (1:1 to 8:1). After 84 hours culture, cells were harvested and pooled for flow cytometer analysis.

### Statistical analyses

Statistical analyses were performed using Prism 7(Graph Pad). Student’s t-test was used to compare the parameters between the two groups. One way ANOVA followed by Tukey’s multiple comparison was used for multi group analyses. *P* values < 0.05 were considered statistically significant.

## Results

**Fig 1:**
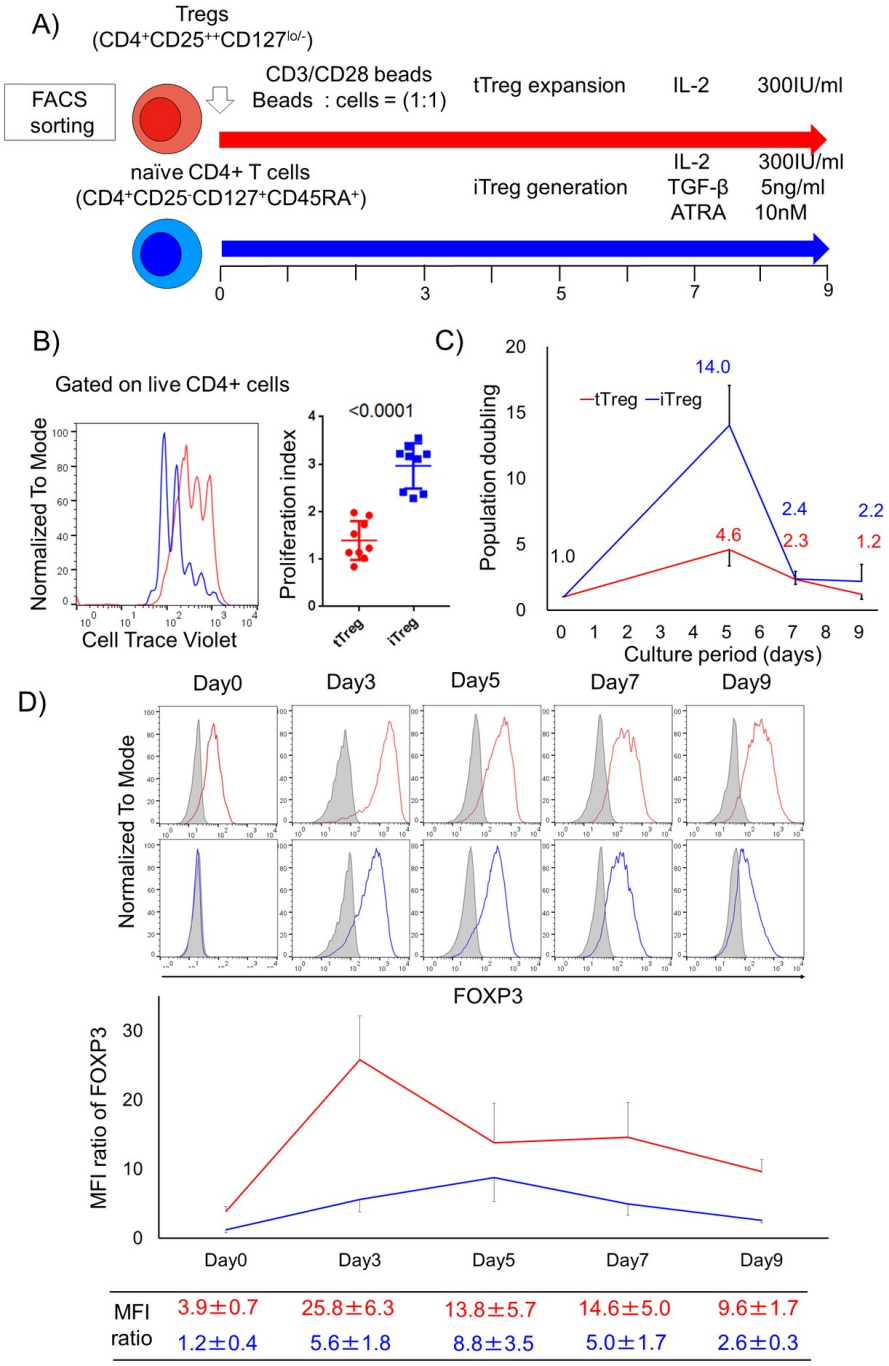
Kinetics of proliferation and FOXP3 expression in human Treg subsets post activation. (A) FACS isolated Tregs and naïve CD4^+^ T conventional cells were cultured in Treg expansion conditions (red) and iTreg generation conditions (blue), respectively. (B) Cells were stained with Cell Trace Violet and their dilution was compared at day 3 post activation in representative histograms and proliferation index that indicates mean frequency of cell division (n = 9 from 6 individual experiments). (C) Line plot data shows population doubling during in vitro culture of 5 different donors from 2 independent experiments. Plotted number and s.d. bars indicate mean population doublings and s.d., respectively. (D) FOXP3 expression by flow analysis over time. Data, mean ± s.d. of at least 5 independent donors for each time points from 6 individual experiments, are expressed as the MFI ratio of FOXP3. The MFI ratio of FOXP3 were calculated as MFI of FOXP3 divided by MFI of isotype control on live CD4^+^ cells.

To determine the metabolic differences between human tTregs and iTregs, we first isolated ex vivo Tregs (tTregs) and naïve CD4^+^ T cells (S1 Fig). tTregs were stimulated with CD3/CD28 antibody beads in the presence of IL-2 [21], while iTregs were induced by culturing naïve CD4^+^ T cells with CD3/CD28 antibody beads in the presence of TGF-β, ATRA and IL-2 [22] (Fig 1A). We observed that iTregs divided more than tTregs by day 3 post activation as assessed by their higher proliferation index (PI) (mean PI, tTreg 1.4±0.4 vs iTreg 3.0±0.5, P<0.001, (Fig 1B). As a result, the mean population doubling was 3 fold higher in iTregs at day 5 compared to tTregs (Fig 1C). Concomitantly, we found that tTregs substantially increased FOXP3 expression upon activation. In contrast, the kinetics of FOXP3 expression in iTreg was transient. FOXP3 was induced by day 3 although not to the same degree as tTregs; reached its maximum around day 5, and decreased by day 7 (Fig 1D).

**Fig 2:**
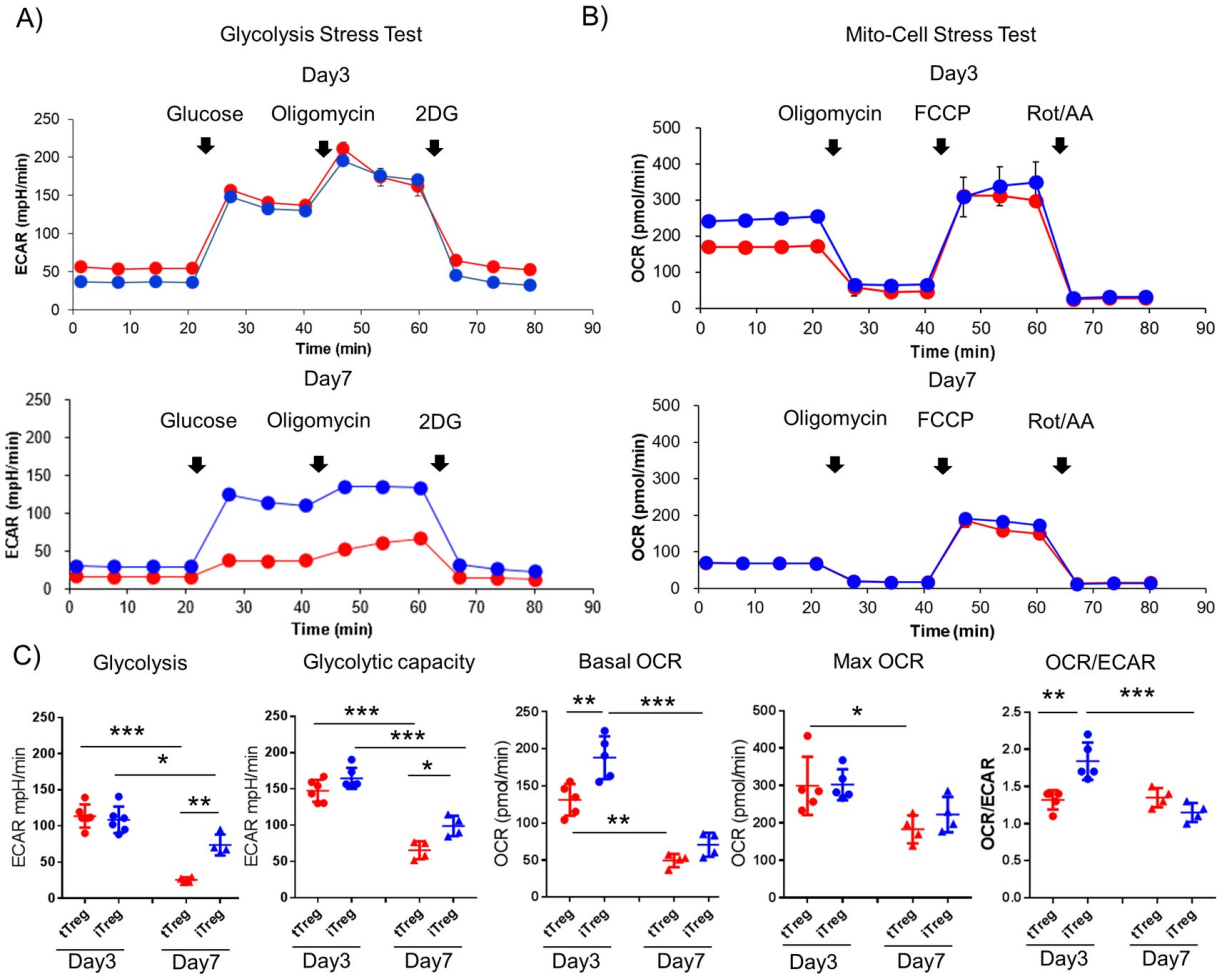
Metabolic traits of in vitro activated human tTregs and iTregs. Real-time measurements of (A) extra cellular acidification rates (ECAR) and (B) oxygen consumption rates (OCR) of activated tTregs (red) and iTregs (blue) were assessed in response to glucose, oligomycin, and 2-deoxy-D-glucose (2DG) (glycolysis stress test, A) and oligomycin, carbonyl cyanide-p-trifluoromethoxyphenylhydrazone (FCCP) and rotenone/ antimycin (mito-cell stress test, B) at day 3 and 7 post activation, respectively. Representative real-time data are shown from 5 individuals for day 3 and 4 individuals for day 7 (mean ±s.d. of n = 3 samples per group). (C) Metabolic profiling data of day3 (left side) and day 7 (right side). Glycolysis and glycolytic capacity from glycolysis stress test and basal OCR, MAX OCR and OCR/ECAR ratio from mito-cell stress test, are plotted with mean line and s.d. bar, respectively. Statistical analysis shown only on significant different data (*P<0.05, **P<0.01 ***P<0.001, One way ANOVA followed by Tukey’s multiple comparison).

Given that activation and proliferation of conventional T cells is dependent upon upregulation of glycolytic activity [11], and murine FOXP3 is known to negatively regulate glycolysis [12, 13, 14], we determined the bioenergetics of the two Treg subsets following activation. Seahorse analyses revealed that on day 3, both human Treg subsets exhibited similar ECAR profiles (Fig 2A). This suggests that the two types of Tregs have equal capacities to engage aerobic glycolysis at day 3 post activation, despite markedly differential expression of FOXP3 (Fig 1D). Moreover, iTregs exhibited a higher basal OCR and consequently an elevated OCR/ECAR ratio, suggesting a greater reliance upon oxidative metabolism (Fig 2B and 2C). Interestingly, by day 7 post-activation, the OCR/ECAR ratio of iTregs decreased, consistent with increased reliance on glycolytic metabolism, while that of tTregs remain unchanged (Fig 2C). To assess the effect of different conditioning regimens, we cultured tTreg in iTreg conditions (i.e. activation in the presence of IL-2, TGF-β and ATRA). Under iTreg skewing, tTregs showed no changes in survival, proliferation, FOXP3 upregulation or their metabolic capacity at 3 days post activation compared to the “usual” activation protocol (i.e. activation in the presence of IL-2 alone) (S2 Fig). These findings suggest that although both human Treg subsets utilize glycolytic metabolism equally during the early phase of activation, their metabolic phenotypes diverge during the expansion phase indicating a distinct temporal metabolic regulation in each of the subsets.

**Fig 3:**
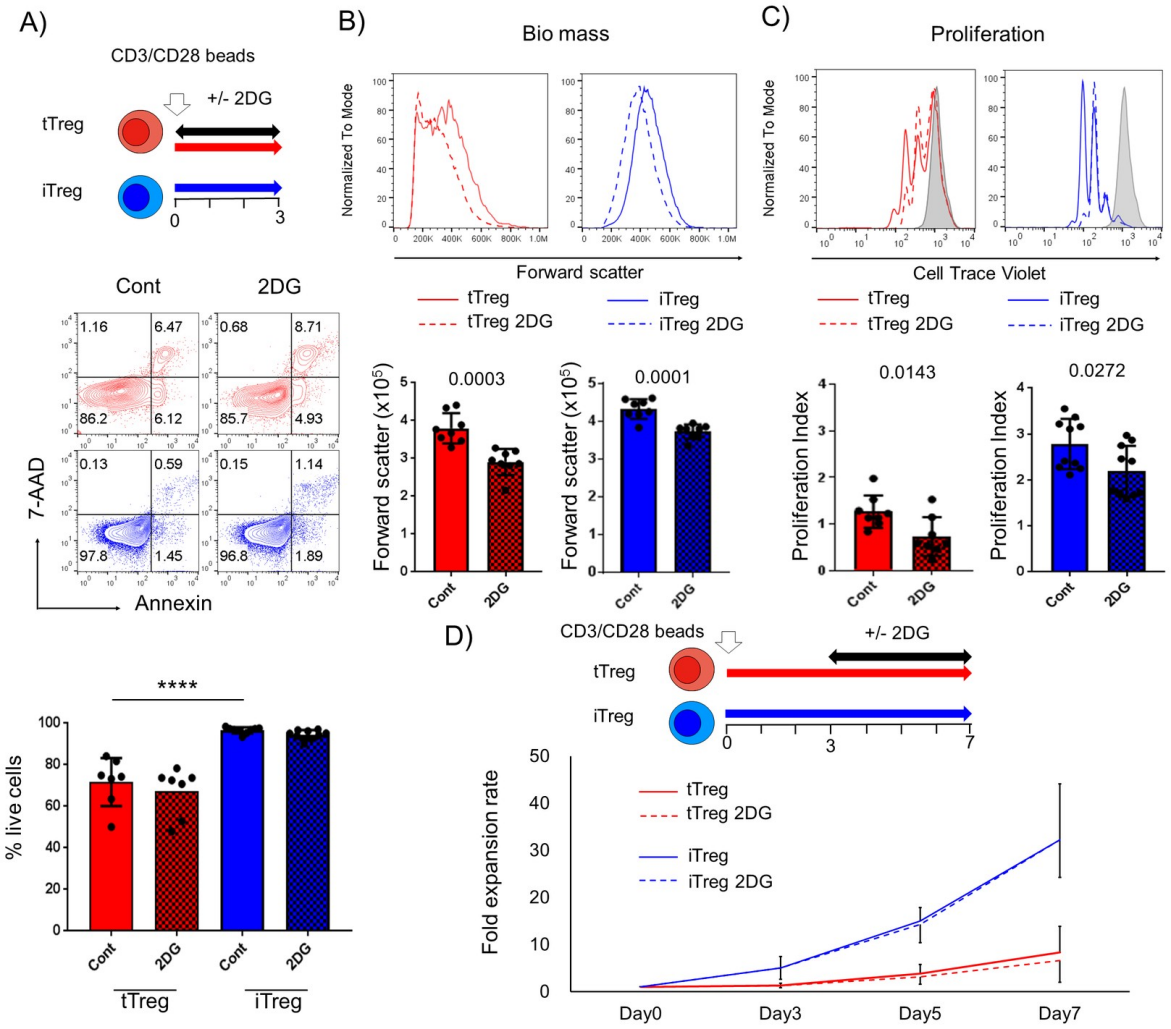
The timing of 2DG treatment has distinct effects on the proliferation of Treg subsets. tTregs (red) and iTregs (blue) were cultured with 0.5mM 2-deoxy-D-glucose (2DG) from onset to day 3 post activation (n = 9). (A) 2DG effect on survival of human Treg subsets was analyzed by annexin-7AAD staining. Impact of Inhibiting glycolysis on (B) cell biomass by forward scatter, and (C) proliferation by Cell Trace Violet dilution assay were assessed. Data are shown as non-treated control (Cont) and treated Tregs (tTreg 2DG or iTreg 2DG) as solid and dot lines in representative histograms, respectively, with isotype control (filled gray). In bar graphs, solid (Cont, non-treated) and checked (2DG, treated) boxes indicate the mean ±s.d. (D) Line plot data show mean fold expansion rate (± s.d.) treated with (dot) or without (solid) 0.5mM 2DG from day 3 post activation (n = 5). Representative data is shown from at least 3 independent experiments. Statistical analyses were performed by Student’s t-test or One way ANOVA followed by Tukey’s multiple comparison (*** P<0.001 is shown only on significant different data in multiple comparison).

Since glycolysis is critical for providing metabolic precursors necessary for cell growth and proliferation for T effectors [24], we sought to determine if this is the case in human Treg subsets by culturing Treg subsets in the presence of 2DG, a glycolytic inhibitor. To determine the appropriate dose of 2DG, we first performed a dose titration of 2DG added at culture initiation (S3 Fig). We observed that tTregs had higher basal levels of apoptosis than iTregs and 0.5mM 2DG did not cause major changes in the survival of either Treg subset (Fig 3A). However, at this concentration, 2DG did reduce the biomass accumulation (assessed by cell size, i.e., forward light scatter), as well as proliferation of both Treg subsets (Fig 3B, 3C and S3 Fig). In particular, tTregs appeared to be more sensitive to glycolytic inhibition than iTregs, as revealed by a significantly higher reduction of the proliferative index of tTregs (mean %, tTreg 45.0±17.8 vs iTreg 22.1±5.8, P = 0.0014) and the ability of the higher concentration of 2DG (1mM) to almost completely abrogate the proliferation of the former population while only partially inhibiting the latter (S3 Fig). We also determined if 2DG treatment had similar effect on Treg proliferation if administered post activation. Surprisingly, addition of 2DG at day 3 did not impair the proliferation of either of Treg subset suggesting that Tregs become insensitive to glycolytic inhibition once activation and division ensue (Fig 3D).

**Fig 4:**
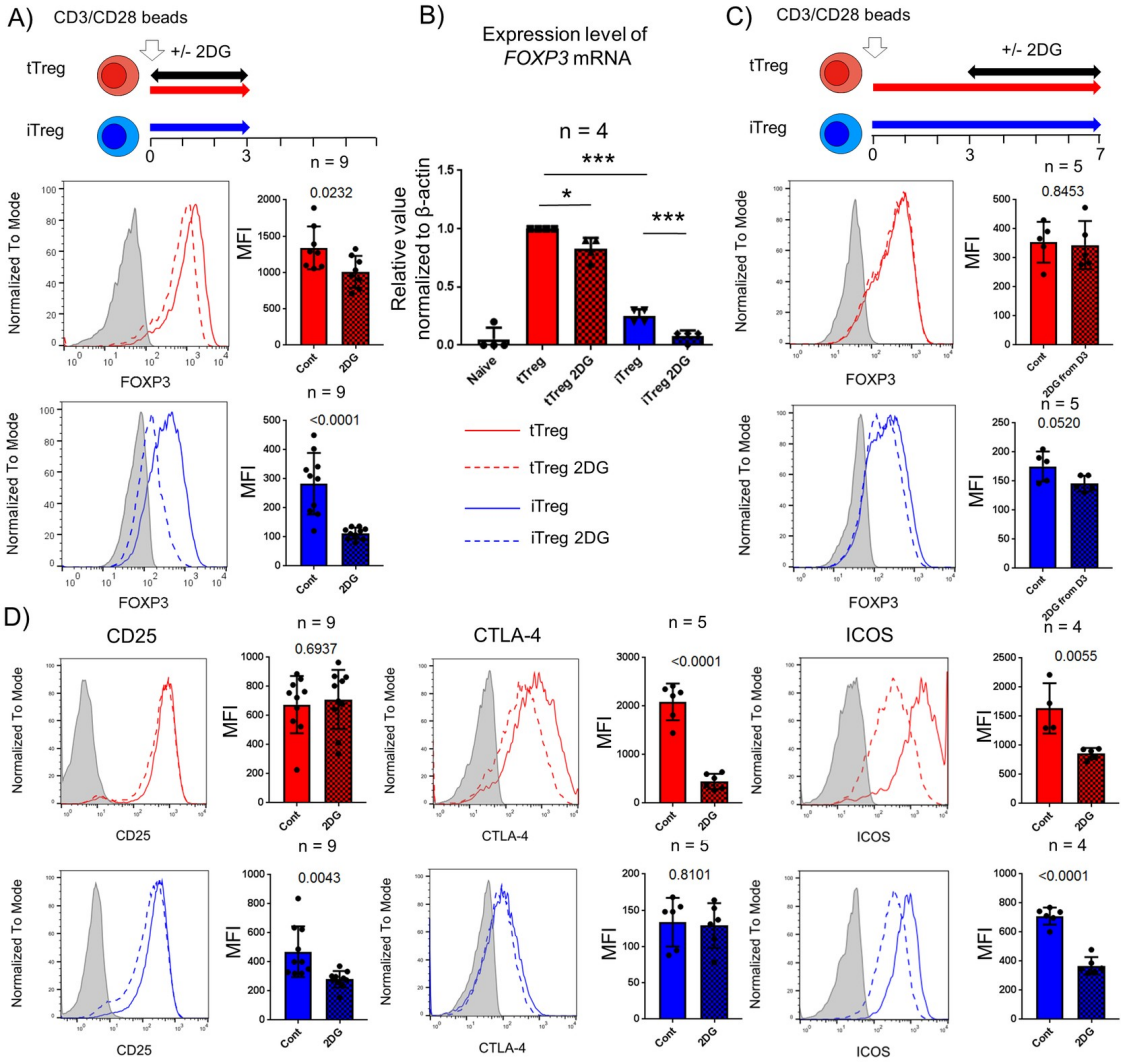
Critical requirement of glucose metabolism in Tregs for optimal expression of functional molecules during initial activation. 0.5mM 2-deoxy-D-glucose (2DG, used to inhibit glycolysis, was added from the onset to day3 (A, B and D), and from 3 to 7 days post activation (C) in tTreg (red) and iTreg (blue). Flow analyses for FOXP3, CD25, CTLA-4 and ICOS were performed gated on live CD4^+^ cells. Data are shown as non-treated control and 2DG treated as solid and dot lines in representative histograms with isotype control staining (filled gray). Solid (non-treated, Cont) and checked (2DG treated, 2DG) boxes with bar graph indicates the mean fluorescence intensity (MFI) ±s.d.. (B) Quantitative PCR analysis of all *FOXP3* mRNA were performed with post sorted naïve T conventional cells (Naïve) and 3 days cultured products (tTreg and iTreg±2DG). Representative data is shown from at least 3 independent experiments. Statistical analyses were performed by Student’s t-test or One way ANOVA following Tukey’s multiple comparison (*P<0.05, *** P<0.001 is shown only on significant different data in multiple comparison).

We next examined the effects of 2DG treatment on the expression of key functional markers including FOXP3 expression and the suppressive capacity of Treg subsets. Firstly, we observed that 2DG treatment at the time of initial stimulation had only a minimal effect on FOXP3 expression in tTregs, whereas by contrast, inhibiting glycolysis in iTreg strongly blocked the induction of FOXP3 (Fig 4A). We also observed decreased mRNA level of *FOXP3* at day 3 post activation in both Treg subsets but more profoundly in iTregs suggesting that the effect of 2DG on FOXP3 expression is regulated at the transcriptional level in both these subsets (Fig 4B). Recently, glycolysis was reported to control FOXP3 induction in human iTregs which were generated via a weak TCR activation system without the addition of TGF-β [19]. Mechanistically, enolase I, a glycolytic enzyme, has been shown to bind to the epigenetic promoter region of *FOXP3* gene to repress transcription of a specific *FOXP3* exon-2 variant [19]. Consistent with this, co-staining with FOXP3 exon2 epitope specific mAbs (D150/E4) and pan FOXP3 mAbs (PCH101) revealed that the FOXP3-exon2 variant was the major component of FOXP3 in both tTregs and iTregs, and that 2DG treatment specifically reduced its expression in iTregs but not tTregs (S4 Fig). Murine studies show that hyperactivation of signaling pathways that enhance glycolysis can result in the destabilization of FOXP3 and lead to Treg lineage instability and dysfunction [14, 16, 25]. To understand the link between glycolysis and FOXP3 maintenance after activation in human Treg subsets, we performed 2DG treatment at day 3 post activation. 2DG treatment after day 3 did not show any effect on FOXP3 expression in either iTregs or tTregs (Fig 4C). Together, these data suggest that although glycolysis is critical for FOXP3 expression during early activation, it is dispensable for its maintenance in activated Treg subsets.

In addition to its effect on FOXP3 expression, glucose metabolism mediated by mTORC1 signaling promotes the expression of Treg functional molecules such as CTLA-4 and ICOS in murine Tregs [17]. 2DG added at the onset of culture, reduced expression of both CTLA-4 and ICOS but not CD25 in tTregs while it reduced ICOS and CD25 expression in iTregs (Fig 4D). Furthermore, addition of 2DG at day 3 post activation decreased only ICOS expression in both Treg subsets but neither CD25 nor ICOS were affected suggesting that Treg subsets have differential glycolytic requirements for optimal expression of these functional molecules that is dependent on the phase of activation (Fig 4D and S5 Fig).

**Fig 5:**
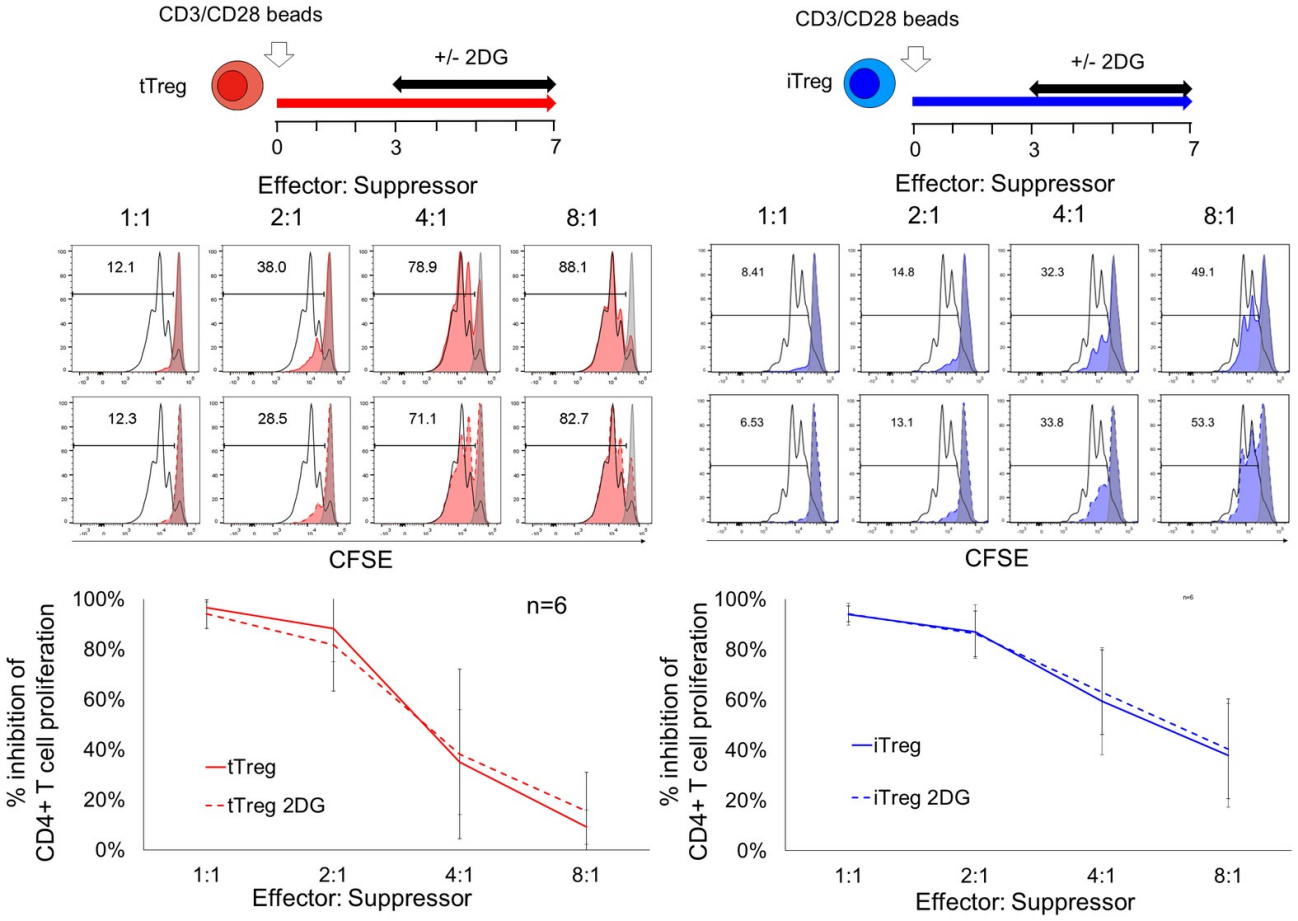
2DG treatment at day 3 did not affect suppressive capability of Treg subsets. Suppression assays were performed with 7 days cultured tTreg (red) and iTreg (blue) after they were rested in 50IU/ml IL-2 medium for an additional 2 days prior to assay. Autologous CD4 enriched T responders were stimulated by CD3/CD28 microbeads at 1:5 bead to cell ratio for 84hrs in absence or presence of suppressors indicated effector to suppressor ratio. Representative histogram gated on live CD4+ responder show the CFSE dilution profiles with stimulation control (open black) and unstimulated control (filled grey). % inhibition of CD4+ T cell proliferation was calculated as %proliferating (stimulated-sample)/ %proliferating cells (stimulated—unstimulated). Representative data is shown from 3 independent experiments of total 6 individual donors with experimental duplicates.

Next, we determined the effect of 2DG treatment on the suppressive capacity of Treg subsets. Given the critical effect of 2DG on FOXP3 expression at initial activation that could compromise the suppression function, we performed suppression assays using 7 day cultured Treg subsets with 2DG treatment from day 3–7. Addition of 2DG at day 3 didn’t alter the suppressive capabilities of both tTreg and iTreg (Fig 5).

**Fig 6:**
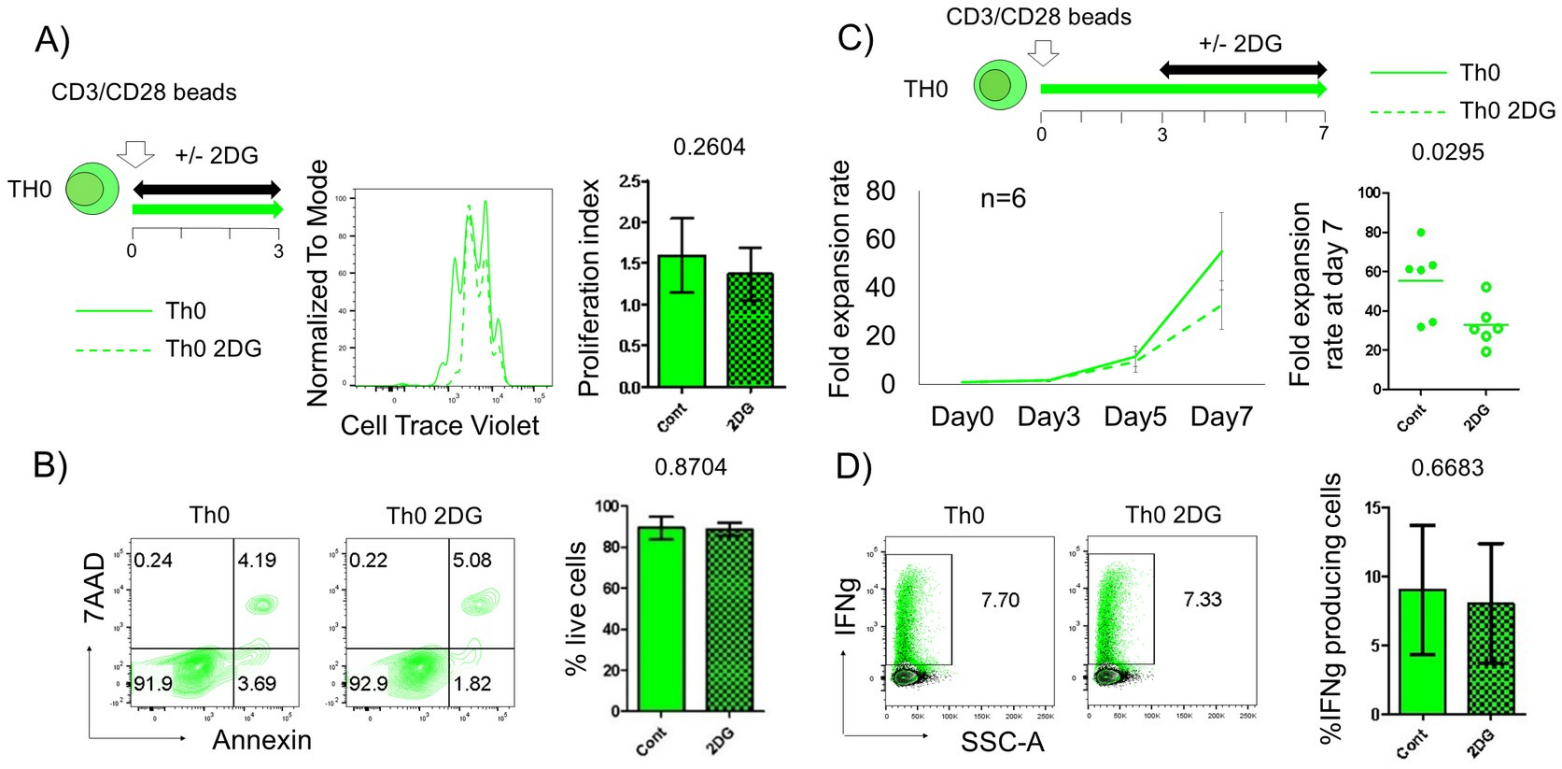
Effect of 0.5mM 2DG on T conventional cells. Th0 cells (green) culturing performed with 0.5mM of 2-deoxy-D-glucose from onset to day 3 post activation (A and B) or from day 3 to day 7 post activation (C and D). Cell proliferation (A) and survival (B) status were analyzed at day3 post activation by cell tracer dilution assay and Annexin-7AAD, respectively. (C) Line plot data show mean fold expansion rate (± s.d.) treated with (dot) or without (solid) 2DG (n = 6). Dots plot show the individual plots of fold expansion rate at day 7 post activation. (D) Representative plots show % IFN gamma producing cells in live Th0 cells treated with or without 2DG. Solid (non-treated, Cont) and checked (2DG treated, 2DG) boxes with bar graph indicates the mean fluorescence intensity (MFI) ±s.d.. Representative data is shown from at least 3 independent experiments of total 6 individual donors. Statistical analyses were performed by Student’s t-test.

Finally, given the temporal insensitivity of 2DG on Treg subset proliferation and functional, we then determined the effect of 2DG on the proliferation of T conventional cells, a potential population that can contaminate Treg expansion cultures. Here we used the low dose (0.5mM) 2DG treatment. This concentration is lower compared to previously reported studies where 2DG effect on human T cells was determined at relatively high dose (1-10mM) [26, 27, 28]. We observed that 2DG treatment at the time of initial stimulation does not show significantly impact proliferation and survival of Th0 cells as observed at day3 post activation (Fig 6A and 6B). This is consistent with previous studies that show that T cells can depend on either glycolysis or oxidative metabolism to fuel initial proliferation [26, 28]. Interestingly, 2DG treatment beginning at day 3 post activation significantly suppressed the expansion of Th0 cells (Fig 6C), while the sparing the Treg populations (Fig 3D). Furthermore, although it has been reported that IFN gamma producing capacity is regulated by glycolysis [26], addition of 2DG treatment at day 3 post activation did not diminish cytokine production in Th0 cells (Fig 6D). Together, these findings suggest that blockade of glucose metabolism post activation diminishes the proliferation of effector cells without impairing their cytokine producing capability.

## Discussion

The successful implementation of Treg therapy (either polyclonal or antigen specific) relies upon efficient Treg proliferation and maintenance of functional stability both in vitro and in vivo. Data from murine studies with metabolic inhibitors demonstrated that differentiation of iTregs require FAO, whereas that of functional T effectors require glycolysis and blocking glycolysis promotes iTreg differentiation [10, 11]. Genetically modified murine models also showed that enhanced glycolysis in Tregs can in fact diminish FOXP3 expression and disrupt their lineage stability [14, 16, 25]. However, it is becoming increasingly evident that this is not a set paradigm. Others and we show that activated ex vivo Tregs (both mice and human) can engage in glycolytic metabolism [18, 29]. In our observations, we found that both human tTreg and iTreg subsets equally engage in glycolysis post activation, but iTregs concurrently show greater utilization of oxidative metabolism potentially to support their higher proliferative capacity. TGF-β containing iTreg skewing conditioning did not affect glycolytic metabolism and proliferation of tTregs. This is in contrast to murine tTregs, where exposure to TGF-β inhibits their glycolytic metabolism and consequently their proliferation [29] indicating a species specific difference of Treg responses to TGF-β.

Interestingly, 2DG treatment at the onset of activation significantly reduced growth and proliferation of both tTregs and iTregs. This is in line with previous observations that 2DG treated ex vivo human Tregs under leptin neutralization decreased ^3^H thymidine uptake and BrdU incorporation [18]. However, at higher concentrations of 2DG (1mM), tTregs showed more pronounced decrease in cell division than iTregs, suggesting that although glycolysis is essential for cell growth in both subsets, tTreg are more sensitive to glycolytic inhibition than iTregs to drive their proliferation upon activation.

In addition to proliferation, mouse Treg-specific deletion of mTORC1 shows that glycolytic lipogenesis is required for optimal expression of the suppressive molecules CTLA-4 and ICOS [17]. More recently, glucokinase, an isomer of hexokinase, via mTORC2 signaling was reported to be required for tTreg migration [30]. Along these lines we observed that 2DG treatment during the onset and later post day 3 activation led to loss of ICOS upregulation in both tTregs and iTregs. This suggests that glycolysis is critical for establishing competent Tregs in the human system as well. Furthermore, we also observed that CTLA-4 expression was significantly downregulated by 2DG treatment during the onset only in tTreg but not iTregs further highlighting the differential sensitivities of tTreg and iTregs to 2DG treatment.

From murine studies, FOXP3 itself can actively diminish glycolysis and promote oxidative phosphorylation [12, 13, 14], while glycolysis controls the induction of functional FOXP3 expression in human Tregs [19]. We observed that 2DG at the onset of activation significantly but modestly decreased FOXP3 expression in tTreg and strongly impaired FOXP3 induction in iTregs. However, inhibition of glycolysis at later stages of activation had little or no effect on either Treg subsets. This is consistent with seahorse analyses that show that at later stages, tTregs downregulate glycolysis and sustain higher FOXP3 levels. These findings suggest that glycolysis is required for FOXP3 expression during early activation in both human Treg subsets but becomes dispensable for FOXP3 maintenance during expansion. At this later activation period, high FOXP3 expression may negatively regulate glycolytic metabolism in tTregs. This indicates a temporal and a reciprocal regulation between glycolysis and FOXP3 expression upon activation in each Treg subsets. Finally, we observed that 0.5mM 2DG treatment at day 3 did not change the suppressive capability of Tregs subsets. This is in contrast to the findings which showed that pretreatment of tTregs with 2DG (48hrs prior to suppression assay) in fact reduced the suppression capacity of Tregs [31]. These differences may be attributed to the difference in dosage of 2DG treatment and differences in the conditioning of Tregs with 2DG between the two culturing systems.

2DG treatment has been reported to predominantly affect cytokine production and expansion in both murine [11, 28] and human [27, 32, 33] T effectors. Our data suggest that the temporal glycolytic requirement of human Tregs and Th0 cells could provide a potential therapeutic window to control the balance between Tregs and T effectors. Furthermore, 2DG treatment as a strategy to inhibit contaminating T effectors at the onset is unfavorable for optimal Treg generation. With the advent of chimeric antigen receptor (CAR) Treg technology and our new understanding of how the choice of CAR signaling domains can impact specific metabolic pathways and enhance their in vivo persistence [34], further understanding the temporal metabolic requirements Tregs and T effectors metabolism could be one of the key determinants to selectively augment Treg activity both in vitro and in vivo.

## Supporting information

S1 Fig Sorting strategy for ex vivo Treg and naïve T conventional cells. Healthy donor PBMCs obtained by leukapheresis were enriched for CD4^+^ T cells followed by FACS sorting. (A) Gating strategies to sort CD4^+^CD25^+^CD127^lo/-^ Tregs and CD4^+^CD25^-^CD127^+^CD45RA^+^ naïve T conventional cells. (B) Purity of post sorted Tregs (red) and naïve T conventional cells (blue) were typically more than 95%. (C) Representative histogram shows FOXP3 expression of both Tregs and naïve T conventional cells with isotype matched control staining (grey filled).
(PDF)

S2 Fig Lack of effect of culturing tTregs in iTreg generating conditions. Ex vivo Tregs were cultured in iTreg generating conditions (green) to compare with typical Treg activating conditions (red). (A) Cell survival, proliferation status and FOXP3 expression were analyzed at day 3 post activation. Data are shown in representative histograms and plotted with mean line and s.d. bar, respectively (n = 5). (B) Real-time measurements of extra cellular acidification rate (ECAR) and oxygen consumption rate (OCR) were assessed in same manner as Fig 2. Representative data are from one of 3 independent experiments. Statistical analysis did not show any significance (Student’s t-test).
(PDF)

S3 Fig Effect of dose titration of 2DG on survival and proliferation in Treg subsets. tTreg (red) and iTreg (blue) culturing performed with the indicated dose of 2-deoxy-D-glucose. Cell survival and proliferating status were analyzed at day3 post activation by annexin-7AAD and cell tracer dilution assay, respectively. Representative data is shown from 2 independent experiments.
(PDF)

S4 Fig Inhibiting glycolysis by 2DG specifically suppresses the expression of FOXP3-exon2 variant in iTregs. tTreg (red) and iTreg (blue) cultured with or without 0.5mM 2-deoxy-D-glucose (2DG) at culture onset and analyzed at post day3 activation. (A) Co-staining analyses with FOXP3 exon2 specific (D150/E4) and all FOXP3 (PCH101) mAbs are shown in representative counter plot with isotype control (gray). (B) Bar graphs show the distribution in fraction I—IV defined by quadrant in counter plot, respectively (n = 5). Statistical analysis shown only on significant data (*P<0.05, One way ANOVA followed by Tukey multiple comparison).
(PDF)

S5 Fig The effect of 2DG treatment at day 3 on functional molecules. 0.5mM 2-deoxy-D-glucose (2DG) was added from 3 to 7 days post activation in tTreg (red) and iTreg (blue). Flow analyses for CD25, CTLA-4 and ICOS were performed gated on live CD4+ cells. Data are shown as non-treated control and 2DG treated as solid and dot lines in representative histograms with isotype control staining (filled gray). Solid (non-treated, Cont) and checked (2DG treated, 2DG) boxes with bar graph indicates the mean fluorescence intensity (MFI) ±s.d.. Representative data is shown from at least 3 independent experiments of total 6 individual donors. Statistical analyses were performed by Student’s t-test.
(PDF)
