# Supplementary material for: Differential effects of 2-deoxy-D-glucose on in vitro expanded human regulatory T cell subsets
Source: PLoS One. 2019 Jun 6;14(6):e0217761. doi: 10.1371/journal.pone.0217761 (PMC6553739; doi:10.1371/journal.pone.0217761)
Supplement: S1 Fig — Healthy donor PBMCs obtained by leukapheresis were enriched for CD4+ T cells followed by FACS sorting. (A) Gating strategies to sort CD4+CD25+CD127lo/- Tregs and CD4+CD25-CD127+CD45RA+ naïve T conventional cells. (B) Purity of post sorted Tregs (red) and naïve T conventional cells (blue) were typically more than 95%. (C) Representative histogram shows FOXP3 expression of both Tregs and naïve T conventional cells with isotype matched control staining (grey filled). (PDF) [file pone.0217761.s001.pdf]

S1 Fig. Sorting strategy for ex vivo Tregs and naïve T conventional cells

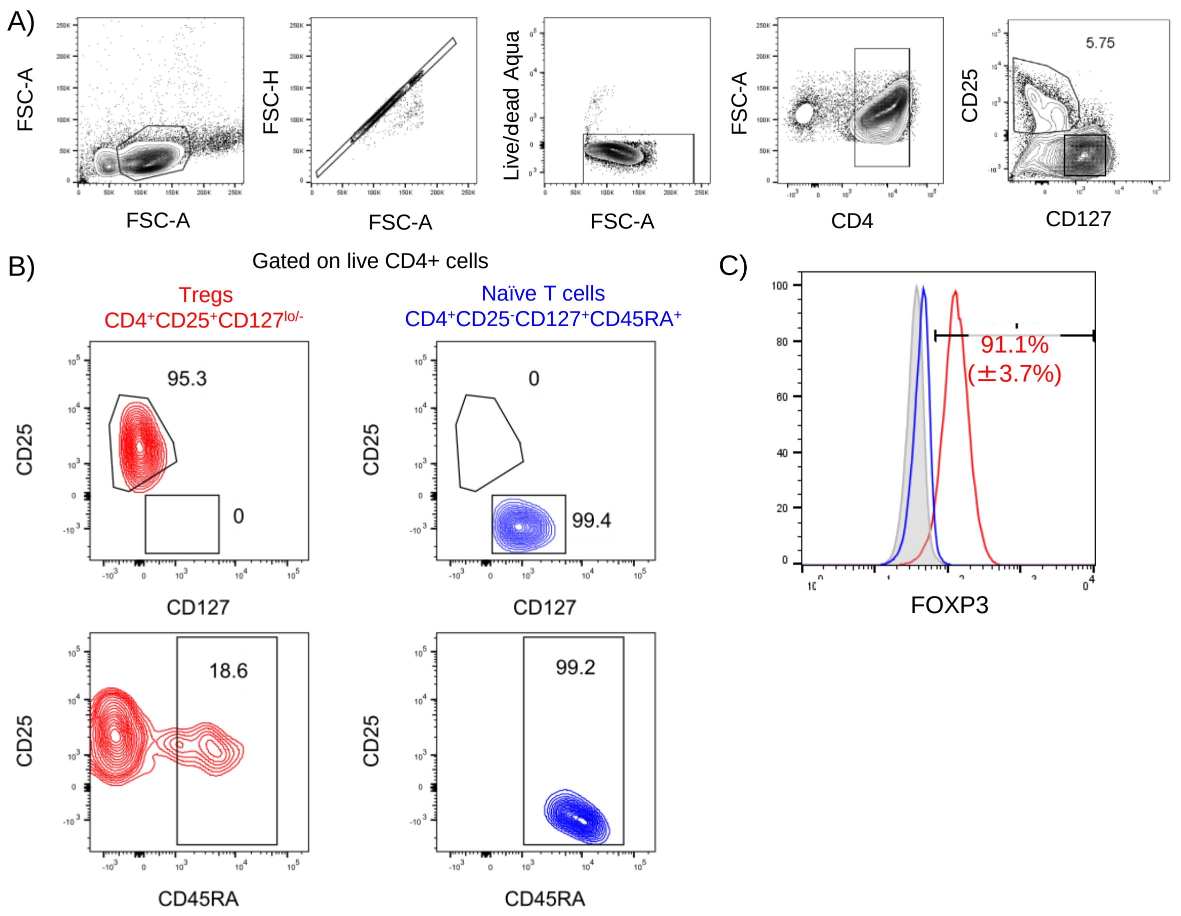

S1 Fig. Healthy donor PBMCs obtained by leukapheresis were enriched for CD4$^+$ T cells followed by FACS sorting. (A) Gating strategies to sort CD4$^+$CD25$^+$CD127$^{lo/-}$ Tregs and CD4$^+$CD25$^-$CD127$^+$CD45RA$^+$ naïve T conventional cells. (B) Purity of post sorted Tregs (red) and naïve T conventional cells (blue) were typically more than 95%. (C) Representative histogram shows FOXP3 expression of both Tregs and naïve T conventional cells with isotype matched control staining (grey filled).
