# Supplementary material for: Differential effects of 2-deoxy-D-glucose on in vitro expanded human regulatory T cell subsets
Source: PLoS One. 2019 Jun 6;14(6):e0217761. doi: 10.1371/journal.pone.0217761 (PMC6553739; doi:10.1371/journal.pone.0217761)
Supplement: S2 Fig — Ex vivo Tregs were cultured in iTreg generating conditions (green) to compare with typical Treg activating conditions (red). (A) Cell survival, proliferation status and FOXP3 expression were analyzed at day 3 post activation. Data are shown in representative histograms and plotted with mean line and s.d. bar, respectively (n = 5). (B) Real-time measurements of extra cellular acidification rate (ECAR) and oxygen consumption rate (OCR) were assessed in same manner as Fig 2. Representative data are from one of 3 independent experiments. Statistical analysis did not show any significance (Student’s t-test). (PDF) [file pone.0217761.s002.pdf]

S2 Fig. Lack of effect of culturing tTregs in iTreg generating conditions

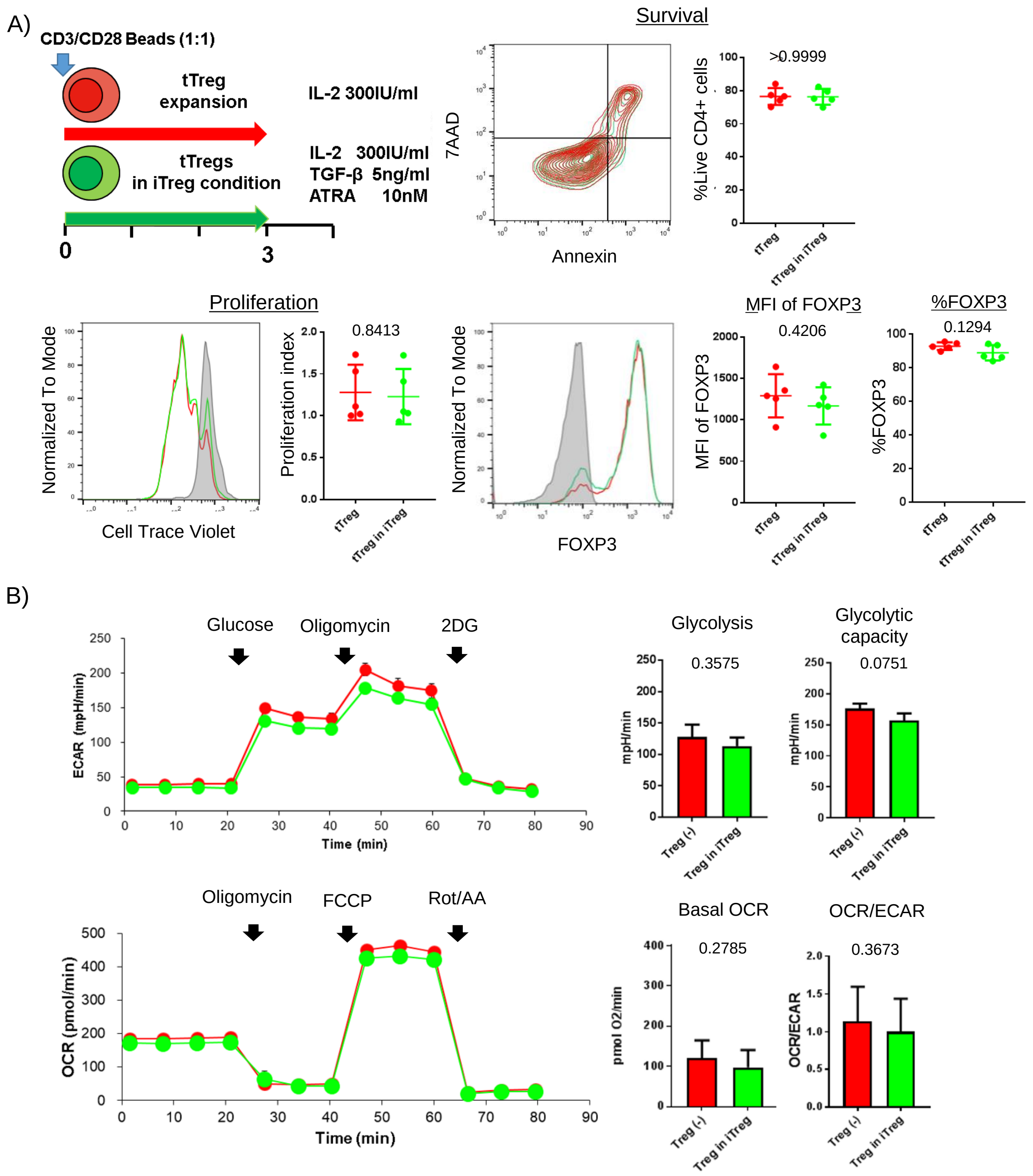

S2 Fig; Ex vivo Tregs were cultured in iTreg generating conditions (green) to compare with typical Treg activating conditions (red). (A) Cell survival, proliferation status and FOXP3 expression were analyzed at day 3 post activation. Data are shown in representative histograms and plotted with mean line and s.d. bar, respectively (n = 5). (B) Realtime measurements of extra cellular acidification rate (ECAR) and oxygen consumption rate (OCR) were assessed in same manner as figure 2. Representative data are from one of 3 independent experiments. Statistical analysis did not show any significance (Student’s t-test).
