# Supplementary material for: Differential effects of 2-deoxy-D-glucose on in vitro expanded human regulatory T cell subsets
Source: PLoS One. 2019 Jun 6;14(6):e0217761. doi: 10.1371/journal.pone.0217761 (PMC6553739; doi:10.1371/journal.pone.0217761)
Supplement: S3 Fig — tTreg (red) and iTreg (blue) culturing performed with the indicated dose of 2-deoxy-D-glucose. Cell survival and proliferating status were analyzed at day3 post activation by annexin-7AAD and cell tracer dilution assay, respectively. Representative data is shown from 2 independent experiments. (PDF) [file pone.0217761.s003.pdf]

S3 Fig. Effect of dose titration of 2DG on survival and proliferation in Treg subsets

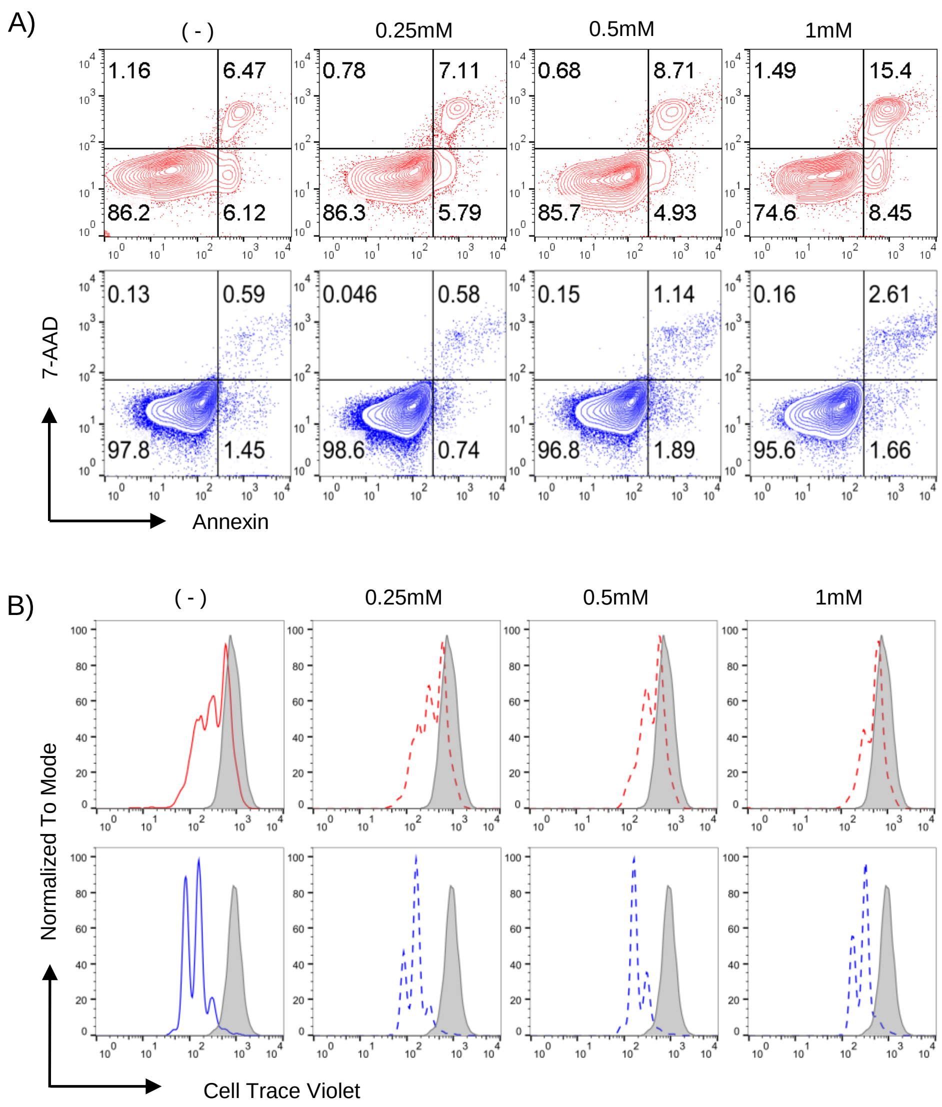

S3 Fig. tTreg (red) and iTreg (blue) culturing performed with the indicated dose of 2-deoxy-D-glucose. Cell survival and proliferating status were analyzed at day3 post activation by Annexin-7AAD and cell tracer dilution assay, respectively. Representative data is shown from 2 independent experiments.
