# Supplementary material for: Differential effects of 2-deoxy-D-glucose on in vitro expanded human regulatory T cell subsets
Source: PLoS One. 2019 Jun 6;14(6):e0217761. doi: 10.1371/journal.pone.0217761 (PMC6553739; doi:10.1371/journal.pone.0217761)
Supplement: S4 Fig — tTreg (red) and iTreg (blue) cultured with or without 0.5mM 2-deoxy-D-glucose (2DG) at culture onset and analyzed at post day3 activation. (A) Co-staining analyses with FOXP3 exon2 specific (D150/E4) and all FOXP3 (PCH101) mAbs are shown in representative counter plot with isotype control (gray). (B) Bar graphs show the distribution in fraction I—IV defined by quadrant in counter plot, respectively (n = 5). Statistical analysis shown only on significant data (*P<0.05, One way ANOVA followed by Tukey multiple comparison). (PDF) [file pone.0217761.s004.pdf]

S4 Fig. Inhibiting glycolysis by 2DG specifically suppresses the expression of FOXP3-exon2 variant in iTregs

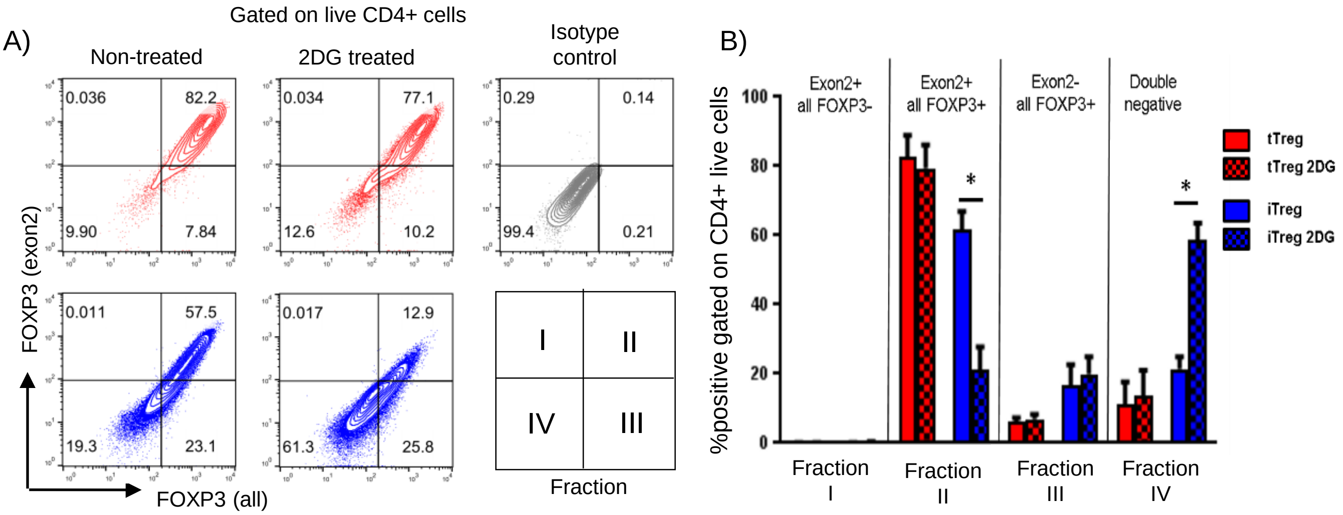

S4 Fig. tTreg (red) and iTreg (blue) cultured with or without 0.5mM 2-deoxy-D-glucose (2DG) at culture onset and analyzed at post day3 activation. (A) Co-staining analyses with FOXP3 exon2 specific (D150/E4) and all FOXP3 (PCH101) mAbs are shown in representative counter plot with isotype control (gray). (B) Bar graphs show the distribution in fraction I - IV defined by quadrant in counter plot, respectively (n = 5 form 3 independent experiments). Statistical analysis shown only on significant data (*P<0.05, One way ANOVA followed by Tukey's multiple comparison).
