# Supplementary material for: Differential effects of 2-deoxy-D-glucose on in vitro expanded human regulatory T cell subsets
Source: PLoS One. 2019 Jun 6;14(6):e0217761. doi: 10.1371/journal.pone.0217761 (PMC6553739; doi:10.1371/journal.pone.0217761)
Supplement: S5 Fig — 0.5mM 2-deoxy-D-glucose (2DG) was added from 3 to 7 days post activation in tTreg (red) and iTreg (blue). Flow analyses for CD25, CTLA-4 and ICOS were performed gated on live CD4+ cells. Data are shown as non-treated control and 2DG treated as solid and dot lines in representative histograms with isotype control staining (filled gray). Solid (non-treated, Cont) and checked (2DG treated, 2DG) boxes with bar graph indicates the mean fluorescence intensity (MFI) ±s.d.. Representative data is shown from at least 3 independent experiments of total 6 individual donors. Statistical analyses were performed by Student’s t-test. (PDF) [file pone.0217761.s005.pdf]

S5 Fig. The effect of 2DG treatment at day 3 on functional molecules

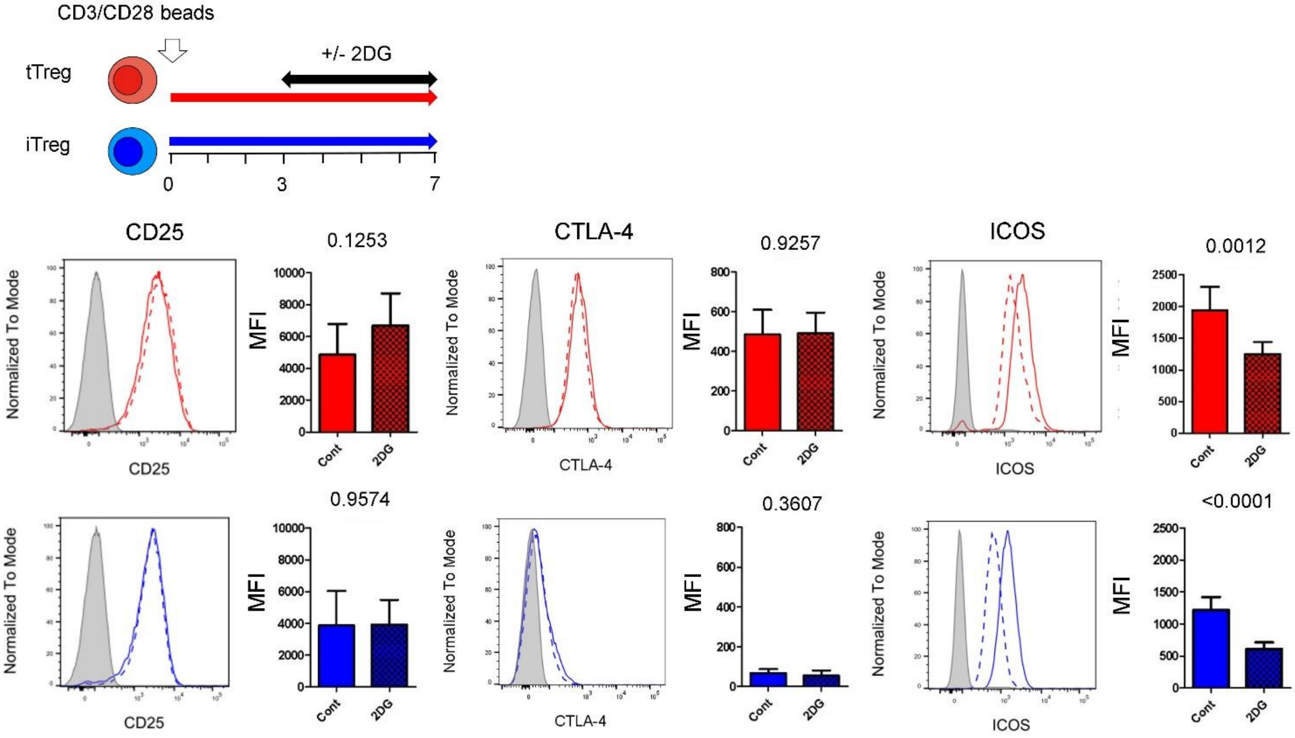

S5 Fig. The effect of 2DG treatment at day 3 on functional molecules. 0.5mM 2-deoxy-D-glucose (2DG) was added from 3 to 7 days post activation in tTreg (red) and iTreg (blue). Flow analyses for CD25, CTLA-4 and ICOS were performed gated on live CD4+ cells. Data are shown as non-treated control and 2DG treated as solid and dot lines in representative histograms with isotype control staining (filled gray). Solid (non-treated, Cont) and checked (2DG treated, 2DG) boxes with bar graph indicates the mean fluorescence intensity (MFI) ±s.d.. Representative data is shown from at least 3 independent experiments of total 6 individual donors. Statistical analyses were performed by Student's t-test.
